# A Modern History of Informed Consent and the Role of Key Information

**DOI:** 10.31486/toj.19.0105

**Published:** 2021

**Authors:** Lydia A. Bazzano, Jaquail Durant, Paula Rhode Brantley

**Affiliations:** ^1^ Department of Internal Medicine, Ochsner Clinic Foundation, New Orleans, LA; ^2^ The University of Queensland Faculty of Medicine, Ochsner Clinical School, New Orleans, LA; ^3^ Institutional Review Board, Division of Academics, Ochsner Clinic Foundation, New Orleans, LA; ^4^ Institutional Review Board, Pennington Biomedical Research Center, Louisiana State University, Baton Rouge, LA

**Keywords:** *Consent forms*, *ethics committees–research*, *informed consent*

## Abstract

**Background:** The concept of informed consent has evolved significantly with regard to both the practice of medicine and research conducted with human volunteers. Yet the process of informed consent used in clinical research and the lengthy consent documents that are difficult to comprehend have been criticized.

**Methods:** We review the history of informed consent as a legal and regulatory concept and the intended impact of the new key information section, a requirement that was introduced in the 2017 revisions to the Common Rule.

**Results:** The key information section is intended to be a concise and focused presentation at the beginning of the informed consent document that facilitates potential participants’ comprehension of the research. However, the lack of regulatory guidance regarding content and length has been problematic. To avoid the risk of noncompliance, many institutions have sought safe harbor by following the limited format guidelines included in the preamble to the revisions to the Common Rule.

**Conclusion:** Research examining formats for the key information section and aids to increasing potential participants’ understanding of a research project should be conducted to ensure that the new regulations achieve the original intent rather than simply lengthening an already lengthy paper document. In addition, the human research protections community should evaluate whether the key information section increases research participants’ understanding of what they will be undertaking in a particular study.

## INTRODUCTION

Informed consent is one of the primary principles on which the framework of protections for human subjects in research is built. In the United States, informed consent was codified in law via the statutes at 45 CFR 46 and 21 CFR 50 of the Code of Federal Regulations, yet the intellectual scaffolding on which it has been built over time has shifted, just as the landscape of human subjects research itself has changed. The most comprehensive modifications to the Common Rule—the Federal Policy for the Protection of Human Subjects—since its adoption in 1991 were enacted in 2018.

The need for improving the process of informed consent has been documented by a wide variety of studies.^1-6^ Empirical research has demonstrated that the informed consent process often fails to provide information in an understandable format to individuals with low health literacy and that the expectation of detailed information recall from a document that is often more than 20 pages is not realistic.^7,8^ To explain how the process of informed consent has evolved over time, how the limitations^9^ of the process developed, and how well these limitations may be addressed by new regulations, we review the intellectual and legal scaffolding of informed consent as it currently exists.

## HISTORY OF INFORMED CONSENT

The concept of informed consent has a relatively short history, beginning with a series of 4 judicial decisions in the early 20th century that laid the foundation for the principle of patient autonomy.^10-12^ These legal decisions began in 1905, with the cases of *Mohr v Williams* and *Pratt v Davis*.^13,14^ Subsequently, 2 additional cases,^15,16^ *Rolater v Strain* and *Schloendorff v Society of New York Hospital*, established and solidified the principle of patient autonomy that ultimately formed the basis of the requirement for informed consent in medicine and research.

In the case of *Mohr v Williams*, the plaintiff, Mrs Anna Mohr, consented to an operation on her right ear; however, once she had been anesthetized, the defendant physician changed the plan of surgery from the right ear to the left after determining that the right ear was not as severely affected by disease as had been expected.^14^ Mrs Mohr's hearing was further impaired by the operation, and she sued the surgeon for assault and battery in changing the laterality of the operation without consent. The Supreme Court of Minnesota agreed that the surgeon should have obtained consent before performing surgery on the opposite ear. Similarly, in the case of *Pratt v Davis*, a 1905 Illinois appellate decision, the plaintiff, Mrs Parmelia J. Davis, had filed suit against her surgeon for battery after he performed a hysterectomy without her consent.^13^ The physician had obtained consent for an earlier operation but admitted to failing to obtain consent for the second procedure and not disclosing the fact that he intended to perform a hysterectomy to treat Mrs Davis's epileptic seizures. The surgeon, Dr Edwin H. Pratt, acknowledged intentionally misleading the plaintiff as to the purpose of the operation, claiming that because Mrs Davis suffered from epilepsy, she was not competent to give her consent or to deliberate intelligently about her situation.^13^ In its decision in favor of Mrs Davis, the appellate court stated,

…under a free government at least, the citizen's first and greatest right, which underlies all others—the fight to the inviolability of his person, in other words, his right to himself is the subject of universal acquiescence, and this right necessarily forbids a physician or surgeon, however skillful or eminent, who has been asked to examine, diagnose, advise and prescribe (which are at least the necessary first steps in treatment and care) to violate without permission the bodily integrity of his patient.^13^

The third case of this early period, *Rolater v Strain*, extended the findings of the legal decisions in the *Mohr* and *Pratt* cases to similar situations in which surgeons performed procedures that the patient had explicitly controverted.^16^ In *Rolater*, the plaintiff sued her surgeon for removing a bone from her foot during an operation that was ostensibly to incise and drain an infection. While the surgeon had obtained consent to perform the procedure to drain an infection, the patient had expressly stated the wish that the surgeon not remove the bones of the foot during surgery, so that the removal constituted a trespass to her person and resulted in the charges of assault and battery. In contrast to the ruling in the cases of *Mohr* and *Pratt*, in the *Rolater* case, the surgeon had obtained the patient's consent before the surgical procedure and performed the surgery on the proper foot. Nevertheless, the Supreme Court of Oklahoma held that the principles of the earlier cases were applicable because the surgeon had not performed the procedure in the manner agreed upon between the physician and patient.

The 1914 case of *Schloendorff v Society of New York Hospital* was the final landmark case that legally established the principle of patient autonomy. The plaintiff, Mary Schloendorff, explicitly stated her wish not to undergo surgery yet was subjected to hysterectomy to remove a fibroid tumor without her consent.^15^ In the ruling, Judge Benjamin Cardozo wrote, “Every human being of adult years and sound mind has a right to determine what shall be done with his own body; and a surgeon who performs an operation without his patient's consent commits an assault, for which he is liable in damages.”^15^

Notably, these landmark cases that established the legal precedent of patient autonomy all had female plaintiffs at a time when women did not have the right to vote in the United States, indelibly intertwining the right of patient autonomy with the right of a woman to consent to procedures on her own body.

Nevertheless, the principle of “informed consent” remained nameless and not legally binding until the term was first publicly recorded in the court documents for the 1957 case *Salgo v Leland Stanford Jr University Board of Trustees*.^17^ The plaintiff in the case, Mr Martin Salgo, had arteriosclerosis of the aorta and underwent a translumbar procedure to evaluate its extent. During the procedure, a contrast agent was injected into his aorta to identify blockages, and the procedure resulted in permanent paralysis of his lower limbs. Mr Salgo sued the university medical center and its chief surgeon for lack of disclosure of this potential risk. In a California appellate court decision, the court directed that each physician must exercise practical insight in completely divulging potential procedural hazards and that physicians are liable for failing to disclose information that a patient would need to make an informed decision regarding medical procedures.^17^ This legal ruling was the first to identify and focus attention on the need to provide the patient with information about the potential benefits and the risks of any medical procedure.

While these cases established the legal framework for and the principle of informed consent, as well as the duty of physicians to obtain informed consent for diagnostic and/or therapeutic medical procedures, the concept of informed consent in human subjects research began to emerge in parallel as a consequence of the investigation of Nazi war crimes at the end of World War II. On August 20, 1947, the trial of 23 physicians and bureaucrats charged with crimes against humanity and war crimes for medical experiments conducted on concentration camp inmates concluded in Nuremberg, Germany.^18^ The verdict of the International Military Tribunal, a trio of American judges empowered under international law adopted by the Allied powers, set forth a series of 10 basic rules for the conduct of human experiments that has become known as the Nuremberg Code.^19^ The Nuremberg Code represents the first explicit attempt to regulate the ethical conduct of research experiments with human subjects and is notable for the emphasis it places on voluntary consent. A section of the ruling entitled “Permissible Medical Experiments” states, “…certain basic principles must be observed in order to satisfy moral, ethical and legal concepts” in human subjects research.^18^ The first of these concepts is the voluntary consent of the human subject. In further statements, the court defined the specific context and meaning for this concept:

This means that the person…should have sufficient knowledge and comprehension of the elements of the subject matter involved, as to enable him to make an understanding and enlightened decision. This latter element requires that, before the acceptance of an affirmative decision by the experimental subject, there should be made known to him the nature, duration, and purpose of the experiment; the method and means by which it is to be conducted; all inconveniences and hazards reasonably to be expected; and the effects upon his health or person, which may possibly come from his participation in the experiment.^18^

This 2-sentence statement distills the major issues in the process of informed consent. First, it highlights that a consenting individual must have sufficient knowledge and comprehension to understand what he or she is agreeing to as part of the research. The word *comprehension* is particularly noteworthy. Second, these statements identify several specific items of information that must be made known to the potential subject of the research.

Subsequent events helped to lay the groundwork for the US regulatory definition of informed consent in human subjects research, a definition that was articulated in federal law with the publication in 1981 of coordinated US Department of Health and Human Services and US Food and Drug Administration (FDA) policies for human subjects research protections as 45 CFR 46,^20^ and the 1991 adoption of Title 45, Public Welfare, Part 46,^21^ Protection of Human Subjects, subpart A, also known as the Common Rule. The historical events that preceded the regulatory definition included the Declaration of Helsinki adopted by the World Medical Association in 1964,^22^ the work of medical ethicist Henry Beecher,^23^ and the public outcry against the 1972 revelation of the Public Health Service Tuskegee Study of Untreated Syphilis in the Negro Male^24^ that was critical to the creation of the National Research Service Award Act on July 12, 1974.^25^ A provision of the Act was the creation of the National Commission for the Protection of Human Subjects of Biomedical and Behavioral Research. Informed by regular deliberations for more than 4 years and several days of intense discussion in 1976, the Commission's final publication of the Belmont Report in 1979 identified basic ethical principles and guidelines regarding the conduct of research with human subjects.^26^

The Belmont Report identified 3 specific concepts critical to the process of informed consent in research: information, comprehension, and voluntariness. The concept of information includes specific elements: “research procedure, their purposes, risks and anticipated benefits, alternative procedures (where therapy is involved), and a statement offering the subject the opportunity to ask questions and to withdraw at any time from the research.”^26^

The report noted that a number of additional items had “been proposed, including how subjects are selected, the person responsible for the research, etc.,”^26^ and also stated,

…the extent and nature of information should be such that persons, knowing that the procedure is neither necessary for their care nor perhaps fully understood, can decide whether they wish to participate in the furthering of knowledge. Even when some direct benefit to them is anticipated, the subjects should understand clearly the range of risk and the voluntary nature of participation.^26^

This statement speaks to another critical contribution of the Belmont Report, the standard of the “reasonable volunteer” in research who may desire information beyond what a reasonable person undergoing medical therapy would desire. In proposing the reasonable volunteer standard, the Belmont Report concept of *information* appears to explicitly extend beyond the accepted extent of disclosures to patients about diagnostic or therapeutic medical procedures.

The second principle articulated in the Belmont Report, Part C: Applications, Informed Consent, was *comprehension*.^26^ Comprehension became one of the significant issues in contemporary thought regarding informed consent and ultimately led to the idea and implementation of a key information section as one of the changes to the Common Rule announced in final form in 2017^27^ and enacted in subsequent years. The first sentence in the Belmont Report section on comprehension states, “The manner and context in which information is conveyed is as important as the information itself.”^26^ Extensive literature, punctuated in 2015 by the Institute of Medicine workshop on informed consent and health literacy,^6^ recognized the need to close this gap in communicating information in a meaningful manner.^5^

## KEY INFORMATION

For a number of reasons, obtaining informed consent for research from human subjects has become a lengthy and complex process, even for relatively simple studies that pose little or no risk to participants. The modern process of obtaining informed consent from individuals interested in participating in research focuses in large part on a written text that documents investigators’ attempts to provide the critical elements of information that have been outlined in 45 CFR 46.116 from its inception. This focus on a lengthy legal document has been roundly criticized because of poor comprehension by potential research participants. For example, a 1983 article in the *Journal of the American Medical Association* concluded, “…little progress has been made in ensuring that the information is comprehensible, understood, and used.”^2^ Such criticisms have continued and strengthened, with evidence demonstrating that consent forms have increased in length and complexity^28^ over time and are typically written at a much higher educational level than appropriate.^29^ Although changes in federal regulations for human subjects research present the hope of improvement in this status quo of a suboptimal informed consent processes, the wording of the regulation permits the investigator to exercise a large amount of flexibility at his/her discretion, pending acceptability to a member or members of the review board.

In the 2017 revisions to the Common Rule, text at §__.116(a)(5)(i) states,

Informed consent must begin with a concise and focused presentation of the key information that is most likely to assist a prospective subject or legally authorized representative in understanding the reasons why one might or might not want to participate in the research. This part of the informed consent must be organized and presented in a way that facilitates comprehension.^27^

Thus, the purpose of presenting “key information” in a “concise and focused” format is to facilitate comprehension. The regulations do not provide a specific definition of key information, although the preamble to the revised rule includes the following list of topics that likely should be included to meet the regulatory intent of the key information requirement: (1) the fact that consent is being sought for research and that participation is voluntary; (2) the purposes of the research, the expected duration of the prospective subject's participation, and the procedures to be followed in the research; (3) the reasonably foreseeable risks or discomforts to the prospective subject; (4) the benefits to the prospective subject or to others that may reasonably be expected from the research; and (5) appropriate alternative procedures or courses of treatments, if any, that might be advantageous to the prospective subject.^27^ The authors state that the lack of specific instructions about the types of information and the length of the concise presentation in the final rule will allow institutions to design informed consents that are tailored to particular research studies and highlight specific fundamental aspects of that research for prospective participants.^27^

While some flexibility is important given the range of human subjects research, extensive flexibility may result in such variation that in practice, the new key information section of the informed consent document may not fulfill the original purpose of facilitating comprehension. On the other hand, many versions of the key information section implemented at various institutions and by researchers include a near-verbatim list of the 5 items listed in the preamble and instruct subjects to read the entire informed consent document for more details. Although this form of the key information section may not be optimally helpful to participants, it may prevail as the most common version because of the limited guidance provided by official sources regarding content.

The Secretary's Advisory Committee on Human Research Protections (SACHRP) provided commentary and response to several queries posed jointly by the Office for Human Research Protections (OHRP) and the FDA regarding key information in attachment C of their letter of November 13, 2018, to the Secretary of Health and Human Services.^30^ In their recommendations, SACHRP advised flexibility to include other elements of consent, or even additional information that is not a required element of consent, if it would assist a prospective subject or legally authorized representative in understanding the reasons why one might or might not want to participate in the research. Regarding providing information about risks, SACHRP recommended not including the full list of risks and benefits because a lengthy list would not aid in a subject's understanding of which risks may be reasonably foreseeable and concerning. In addition, discomforts and inconveniences associated with participation may be important key information for some studies. However, SACHRP specifically noted that from a compliance perspective, the 5 elements listed in the preamble make them a safe harbor of sorts, but that simply following this format may not be in keeping with the intent of the regulatory change.^30^ In the same letter, SACHRP notably recommends that

…OHRP specifically state that diverging from the preamble suggestions of key information would not incur a compliance risk as long as the full consent document meets the requirements of the regulations. This is critical to encourage the development of creative and potentially better approaches to presenting key information and to improvement of the consent form and process as a whole.^30^

A statement from OHRP permitting divergence from the preamble suggestions would reassure the research community as a whole and potentially allow for creative and effective key information sections.

Letters and commentaries from the research ethics community have expressed both enthusiasm and concern regarding the addition of the key information section.^31^ In a 2019 article, Nancy King wrote that the requirement for the key information section has potentially shifted the process of informed consent into a more genuinely patient-centered exchange of information, but she also pointed out the conundrum of “compliance-vs.-flexibility” and the fact that uncertainty is particularly uncomfortable in the regulated research community.^31^ In a different commentary, Mark Yarborough also recognized the key information requirement as a critical opportunity to improve the process of informed consent but more specifically through its potential to create new standards for disclosure.^32^ In cataloging the proposed changes to informed consent under the revised rule, Jeremy Sugarman noted that the revised rule retains the focus on a traditional written document.^33^ In contrast, Kraft and colleagues called for improving the consent process by offering reasons why some enroll in research and other choose not to enroll.^34^

## CONCLUSION

The OHRP and other federal agencies issued little guidance other than what is provided in the preamble and no examples of key information sections that would comply with the new regulations prior to the implementation of the 2018 revisions to the Common Rule on January 19, 2019. Flexibility in the presentation of the key information is appropriate if sufficient information is presented in a manner that facilitates comprehension. However, the current state of uncertainty—lack of assurance that flexibility or creativity in designing the key information section is not associated with a risk of noncompliance—leaves many institutions in the position of seeking safe harbor by following the format presented in the preamble. Empirical research examining formats and aids to increasing understanding should be conducted to ensure that the new regulations achieve the original intent rather than lengthening an already lengthy paper document. In addition, the human research protections community should collect and examine evidence to determine if the key information requirement increases research participants’ understanding of what they will be undertaking in a particular study.

## Notes

This article meets the Accreditation Council for Graduate Medical Education and the American Board of Medical Specialties Maintenance of Certification competencies for Medical Knowledge and Professionalism.
